# Acute respiratory distress syndrome and acute renal failure from *Plasmodium ovale* infection with fatal outcome

**DOI:** 10.1186/1475-2875-12-389

**Published:** 2013-11-04

**Authors:** Yee-Ling Lau, Wenn-Chyau Lee, Lian-Huat Tan, Adeeba Kamarulzaman, Sharifah Faridah Syed Omar, Mun-Yik Fong, Fei-Wen Cheong, Rohela Mahmud

**Affiliations:** 1 Tropical Infectious Disease Research and Education Center (TIDREC), Department of Parasitology, Faculty of Medicine, University of Malaya, 50603 Kuala Lumpur, Malaysia; 2 Sunway Medical Centre, Bandar Sunway, 46150 Petaling Jaya, Selangor, Malaysia; 3 Department of Medicine, Faculty of Medicine, University of Malaya, 50603 Kuala Lumpur, Malaysia

**Keywords:** *Plasmodium ovale curtisi*, Imported malaria, Acute respiratory distress syndrome, Acute renal failure, Death

## Abstract

**Background:**

*Plasmodium ovale* is one of the causative agents of human malaria. *Plasmodium ovale* infection has long been thought to be non-fatal. Due to its lower morbidity, *P. ovale* receives little attention in malaria research.

**Methods:**

Two Malaysians went to Nigeria for two weeks. After returning to Malaysia, they fell sick and were admitted to different hospitals. *Plasmodium ovale* parasites were identified from blood smears of these patients. The species identification was further confirmed with nested PCR. One of them was successfully treated with no incident of relapse within 12-month medical follow-up. The other patient came down with malaria-induced respiratory complication during the course of treatment. Although parasites were cleared off the circulation, the patient’s condition worsened. He succumbed to multiple complications including acute respiratory distress syndrome and acute renal failure.

**Results:**

Sequencing of the malaria parasite DNA from both cases, followed by multiple sequence alignment and phylogenetic tree construction suggested that the causative agent for both malaria cases was *P. ovale curtisi*.

**Discussion:**

In this report, the differences between both cases were discussed, and the potential capability of *P. ovale* in causing severe complications and death as seen in this case report was highlighted.

**Conclusion:**

*Plasmodium ovale* is potentially capable of causing severe complications, if not death. Complete travel and clinical history of malaria patient are vital for successful diagnoses and treatment. Monitoring of respiratory and renal function of malaria patients, regardless of the species of malaria parasites involved is crucial during the course of hospital admission.

## Background

Acute respiratory distress syndrome (ARDS) is one of the severe complications of malaria [1]. ARDS in falciparum malaria has been intensively studied [2-4]. However, ARDS is not restricted solely to *Plasmodium falciparum* infection. This potentially grave complication has also been reported in malaria caused by *Plasmodium vivax*, *Plasmodium malariae*, *Plasmodium knowlesi* and *Plasmodium ovale*[5-12]. Due to its limited geographical distribution [13], as well as the much lower morbidity [14], *P. ovale* has been overshadowed by other human malaria parasites in the field of medicine and medical research. Nevertheless, recent studies have shown that ovale malaria is caused by two genetically distinct subspecies, *P. ovale curtisi* and *P. ovale wallikeri*[15-18].

In this report, two cases of *P. ovale* infection acquired from the same location were presented. Both cases ended with different outcome, and two interesting turning points were ARDS complication and acute renal failure.

## Methods

### Case presentation

Two Malaysian acquaintances (patients A and B) went to Victoria Island, Nigeria together for a two-week working trip. Lariam® (mefloquine) was used as anti-malarial prophylaxis for the trip. They fell sick after returning to Malaysia and were admitted to different hospitals. Their cases are presented as follows:

### Case A (Isolate MAL-2)

About two months after the trip to Nigeria, patient A (52-year-old Chinese male) was admitted to a hospital due to five consecutive days of fever with chills and rigours. He was jaundiced, anorexic and febrile with body temperature of 37.7°C upon admission. He had mild cough, blood pressure of 110/66 mm Hg, pulse rate of 98 beats per minute (BPM) with peak bilirubin level of 45 μmol/L and hepatosplenomegaly. His lung examination was normal. His urine was tea-coloured. Ultrasound study confirmed the findings of hepatosplenomegaly with signs of chronic cholecystitis and cholelithiasis. Initial haematological investigation showed that he was thrombocytopaenic (37,000/μl) with normal white blood cell (WBC) count (5,800 cells/μl) and haemoglobin level of 13.9 g/dL. He had not travelled to any other places after the trip to Nigeria. The patient had a past history of malaria for three times. The last episode of malaria was six months prior to present admission. However, the species of malaria parasites for the previous malaria episodes was not known. The patient also had an underlying condition of hypertension. Besides, he was a heavy alcohol consumer. Clinical findings on patient A upon admission are summarized in Table 1.

**Table 1:** Summary of initial clinical findings on patient A and B upon admission

**Test (unit)**	**Patient A**	**Patient B**
**{normal range}**	**(Isolate MAL-2)**	**(Isolate MAL-1)**
**Blood Pressure (mmHg)** {90/60 - 130/80}	110/66	102/55
**Pulse rate (BPM)** {60 - 100}	98	60
**Parasitemia (%)**	0.10^#^	0.18
**Haemoglobin (g/dL)** {male: 13.5 - 17.5}	13.9	12.4
**TWBC (× 10**^**3**^**cells/μl)** {4.5 - 11.0}	5.8	3.1
**Platelet (× 10**^**3**^**/μl)** {150.0 - 450.0}	37.0	65.0
**Serum creatinine (μmol/L)** {60 - 110}	82.0	107.0
**Serum urea (mmol/L)** {2.5 - 6.4}	9.0	6.5
**Total serum bilirubin (μmol/L)** {<17}	45.0	16.0
**AST (IU/L)** {15.0 - 37.0}	47.0	47.0
**ALT (IU/L)** {30.0 - 65.0}	29.0	39.0
**Random plasma glucose (mmol/L)** {<11.1}	6.4	9.5

Values in {} indicate the normal range of the respective event investigated, corresponding to the age and gender of patient.

TWBC = total white blood cell count; AST = aspartate aminotransferase; ALT = alanine aminotransferase.

Value marked with “#” was not obtained upon patient’s admission to the hospital.

Patient A was treated immediately for cholecystitis with intravenous (IV) ceftriaxone 2 g daily and IV metronidazole 500 mg thrice daily by the attending gastroenterologist. However, his fever and thrombocytopaenia persisted, and WBC count dropped progressively. On day 5 of admission, blood smears were prepared and examined under the microscope. “*Plasmodium vivax*-like” parasites were found with parasitaemia of 0.10%. Further microscopic examination by a referral diagnostic centre subsequently indicated that this was a mono-infection of *P. ovale*. This was confirmed with nested PCR technique using primers developed from the 18S ribosomal RNA (18S rRNA) gene as applied by previous reports [19-21], coupled with sequencing analysis using Basic Local Alignment Search Tool (BLAST) [22]. Meanwhile, bacteriological culture diagnoses from patient’s blood samples were negative.

He was treated with a course of six doses of Riamet® (artemether and lumefantrine), four tablets per dose, and primaquine for two weeks. Patient A responded well to the anti-malarial treatment clinically and biochemically. Patient’s parasitaemia dropped to 0.06% the following day. Malaria parasites were cleared in less than 48 hours after initiation of Riamet® treatment. He was discharged well on day 8 of hospitalization. He remained well without relapse of malaria throughout his medical follow-up of 12 months with the hospital.

### Case B (Isolate MAL-1)

Around six months after the trip to Nigeria, patient B (59-year-old Chinese male) fell sick and went to a private hospital. He was then referred to a tertiary referral hospital. Upon admission to the referral hospital, he gave a history of intermittent fever with rigours, myalgia and nausea for ten days. His blood pressure upon admission was 102/55 mm Hg, with pulse rate of 60 BPM. Plasma glucose level was 9.5 mmol/L. Jaundice and hepatosplenomegaly were not detected. He was alert and conscious. His lung examination was normal. He made a one-day-trip to Kota Kinabalu, Sabah, three months before the admission. He had no known medical illness and no known history of acquiring malaria. Initial haematological investigation revealed that he was thrombocytopaenic (65,000/μl) with low WBC count (3,100 cells/μl) and haemoglobin level of 12.4 g/dL. Malaria parasites were detected in his blood, with parasitaemia of 0.18%. The species was identified as *P. ovale*, which was further ascertained with nested PCR as mentioned in the previous section. Clinical findings on patient B upon admission are summarized in Table 1, and more clinical details on patient B throughout his hospital stay is available in Table 2.

**Table 2:** Clinical details on patient B (Isolate MAL-1) throughout his hospital stay

**Day**	**BP (mmHg)**	**Hb (g/dL)**	**Plt (×10**^**3**^**/μl)**	**TSB (μmol/L)**	**AST (IU/L)**	**ALT (IU/L)**	**SCr (μmol/L)**	**Blood bacteriological & fungal culture**	**Anti-malarials**	**Antibiotics**	**Additional notes**
**1**	102/55	12.4	65	16	47	39	100	N/A	N/A	N/A	PR 60 BPM; SPO_2_ 97% RA; loss of appetite; Lungs: clear
**2**	92/52	N/A	N/A	N/A	N/A	N/A	107	N/A	Chloroquine	N/A	PR 70 BPM; Lungs: clear;
									Primaquine		Dengue IgM negative
**3**	118/66	N/A	107	13	88	71	112	N/A	Chloroquine	N/A	PR 70 BPM; SPO_2_ 98% RA;
									Primaquine		Lungs: minimal basal crepitations
**4**	106/60	10.5	120	13	50	90	101	Negative	Artesunate	Ceftriaxone	PR 66 BPM; Haemoptysis, epistaxis, shortness of breath; CXR: bilateral haziness
									Quinine		
**5**	115/56	11.2	170	8	56	62	114	N/A	Artesunate	Ceftriaxone	Blood smear: negative for malaria parasites; Transferred to ICU
**6**	104/50	9.4	183	6	58	43	139	Negative	Artesunate	Tazocin®	Blood smear: negative for malaria
**7**	95/56	10.3	197	9	46	42	215	Negative	Artesunate	Tazocin®	Blood smear: negative for malaria
**8**	102/70	9.4	178	8	88	43	291		Artesunate	Tazocin®	Blood smear: negative for malaria; respiratory acidosis
									Primaquine		
**9**	143/64	9.0	184	7	49	39	297	Negative	Artesunate	Tazocin®	Blood smear: negative for malaria
**10**	170/60	8.5	199	10	54	48	316	N/A	Artesunate	Tazocin®	Blood smear: negative for malaria
**11**	165/68	8.1	231	8	99	81	301	N/A	N/A	Tazocin®	Ferritin blood test: 2,118 ng/ml
**12**	200/78	8.1	288	13	128	148	309	Negative	N/A	Vancomycin	
										Imipenem	
**13**	160/60	6.8	311	16	137	190	392	N/A	N/A	Vancomycin	Hemolysis screening: negative;
										Imipenem	CXR: worsening
**14**	170/63	9.1	265	13	47	106	408	N/A	N/A	Vancomycin	
										Imipenem	
**15**	135/51	9.5	270	22	65	74	472	Positive for *Enterobacter cloacae*	N/A	Vancomycin	Rigours; acidosis (resp. & met.)
										Imipenem	ABG: pH 7.128; pCO_2_ 59.5 mmHg;
											SLED 4 hours
**16**	111/45	9.1	225	19	43	51	413	N/A	N/A	Vancomycin	CVVHD
										Imipenem	
**17**	134/57	7.5	179	29	109	65	424	N/A	N/A	Vancomycin	Seizures; SLED 8 hours
										Meropenem	
**18**	135/55	7.3	178	30	38	40	320	N/A	N/A	Meropenem	Gentle HD 4 hours
**19**	130/56	9.0	204	34	40	30	404	N/A	N/A	Meropenem	Gentle HD 4 hours
**20**	147/61	8.6	207	25	43	26	403	N/A	N/A	Meropenem	Severe resp. acidosis; met. acidosis; gentle HD 4 hours
**21**	140/54	N/A	N/A	N/A	N/A	N/A	427	N/A	N/A	Meropenem	Gentle HD 3 hours
**22**	102/48	9.3	166	39	296	119	401	N/A	N/A	Meropenem	Worsening hypoxia; hypotensive; Gentle HD 6 hours
**23**	N/A	N/A	N/A	22	136	109	314	N/A	N/A	N/A	Asystole; death

N/A = not available; BP = blood pressure; Hb = haemoglobin; Plt = platelet; TSB = total serum bilirubin; AST = aspartate aminotransferase; ALT = alanine aminotransferase; SCr = serum creatinine; BP = blood pressure; BPM = beats per minute; PR = pulse rate; SPO_2_ = blood oxygen saturation; RA = room air; IgM = immunoglobulin M; CXR = chest X-ray; ICU = intensive care unit; resp. = respiratory; met. = metabolic; ABG = arterial blood gas; pCO_2_ = partial pressure of carbon dioxide; SLED = sustained low efficiency dialysis; CVVHD = continuous veno-venous haemodialysis; HD = haemodialysis.

Anti-malaria therapy course of chloroquine (chloroquine phosphate 150 mg base) and primaquine (30 mg) was started on patient B. However, he was still febrile (38.4°C) 24 hours later. On day 3 of the admission, patient B developed loose stools and lung examination revealed fine basal crepitations. Blood smear examination showed that the malaria parasites were not cleared. In the morning of day 4, he complained of feeling breathless and lethargic. His body temperature surged to 39.2°C. With presence of basal crepitations, an initial diagnosis of pneumonia was made and IV ceftriaxone was added into the course of treatment.

However, later that day in the afternoon, the patient developed worsening dyspnea, haemoptysis, and subsequently epistaxis. Chest X-ray examination showed bilateral haziness up to the upper zone, which was suggestive of pulmonary haemorrhage. Haematological investigation showed that his platelet count was 120,000/μl and the malaria parasite load was reduced to 0.03%. Despite the lowering of parasitaemia, patient B progressed into respiratory failure. He was intubated and ventilated. His anti-malarial treatment was changed to IV quinine 850 mg (1 dose) and subsequently to IV artesunate 160 mg (for 7 days). Furosemide was given to the patient for presumed pulmonary oedema.

On day 5 of the admission, malaria parasites were completely cleared. However, patient was still febrile with temperature of 40.8°C. He was transferred to intensive care unit. On day 6, patient B was oliguric. Increased level of creatinine and worsening of respiratory acidosis were noted. The patient had developed acute kidney injury (AKI) secondary to overwhelming sepsis. Hypotensive episodes were encountered. Therefore inotropic support was initiated. His antibiotic regime was changed to IV Tacocin® (tazobactam and piperacilin).

On day 12, patient B was still febrile with little improvement on his lung function. He was still dependent on the ventilator. IV Tacocin® was replaced by IV vancomycin and imipenem empirical treatment. All the five sets of bacteriological blood cultures and one set of fungal culture requested earlier on different days came back as negative. Nevertheless, dialysis was started on day 15 due to oliguric AKI with worsening acidosis (mixed respiratory and metabolic). Dialysis was performed on daily basis till the end of his life.

On day 17, bacteriological culture from patient’s blood sample that was collected on day 15 was positive for *Enterobacter cloacae*, which was sensitive to carbapenems. Hence, the imipenem therapy was continued. However, due to epileptic seizures suffered by the patient on day 17, the antibiotic regime was subsequently replaced with meropenem. A computerized tomography (CT) scanning on the patient’s brain showed no abnormality. Another thorax CT scanning showed extensive bilateral lung consolidation and loculated pleural effusion. His fever persisted, and he was on prolonged ventilation with difficulty in weaning off. His condition continued to deteriorate. He went into recalcitrant atrial fibrillation on day 22 and required tripleinotropic support. Further blood bacteriological and fungal cultures were all negative. On the 23^rd^ day of hospital admission, he went into asystole and succumbed to *P. ovale* infection with ARDS, acute renal failure, metabolic acidosis, and nosocomial sepsis.

## Consent

Consent was granted by patient/ patient’s family for the publication of these case reports.

### Post-clinical analysis and interpretation

**Figure 1:**
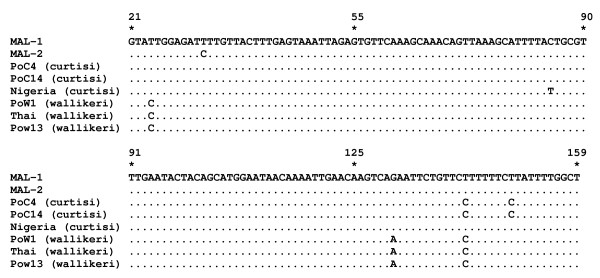
**Multiple sequence alignment of short segments from 18S rRNA gene sequences of *Plasmodium ovale *spp. isolates.** The isolate MAL-1 (GenBank accession number KF192072) and isolate MAL-2 (GenBank accession number KF192073) in this report were indicated as *P. ovale curtisi*, as shown by the high nucleotide sequence similarity shared between these two isolates and gene sequences of other *P. ovale curtisi* isolates (GenBank accession number JF894403, JF894405, L48986) that were available in GenBank. Gene sequences of *P. ovale wallikeri* from GenBank (GenBank accession number JF894407, JF894410) were used in this multiple sequence alignment as well. Position of nucleotides is based on sequences of isolate MAL-1.

**Figure 2:**
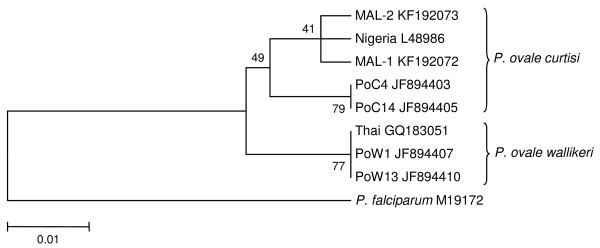
**Phylogenetic tree based on a short segment of 18S rRNA gene sequences of *Plasmodium spp. *isolates.** Isolate MAL-1 (accession number KF192072) and isolate MAL-2 (accession number KF192073) in this report clustered with *P. ovale curtisi* isolates, clearly indicating that these two cases were caused by *P. ovale curtisi*. The tree is constructed using the Neighbor-Joining method (bootstrap = 1000). GenBank accession number is given after each isolate’s name. The 18S rRNA gene sequences of P.falciparum isolate (GenBank accession number M19172) was used as outgroup.

*Plasmodium ovale* is not found locally in Malaysia. Alignment of short segments from 18S rRNA gene sequences of these two cases, i.e. isolate MAL-1 (GenBank accession number KF192072) and isolate MAL-2 (GenBank accession number KF192073) with 18S rRNA gene sequences of other isolates of *P. ovale* spp. that were available in GenBank (accession number L48986, JF894403, JF894405, GQ183051, JF894407, JF894410, M19172) were conducted. The multiple sequence alignment showed that the aetiological agents of the two cases in this case report were *P. ovale curtisi* (Figure 1). The clustering of these two isolates with *P. ovale curtisi* can be visualized clearly via a simple phylogenetic tree constructed using neighbour-joining method (bootstrap = 1000) as described previously [23] (Figure 2).

A case of ovale malaria imported from Nigeria was previously reported in Malaysia [24], and these are another two imported cases of *P. ovale* infection. Unlike the previous case report, which involved a Nigerian student (imported patient) who showed no complications [24], both cases reported here involved Malaysians that acquired malaria from a trip to Nigeria (imported infection), and one showed severe complications that were ultimately fatal. Interestingly, both patients had taken anti-malarial prophylaxis during their trip to Nigeria. Nevertheless, they still contracted malaria. This may be due to usage of the prophylactic drugs without properly following the instructions or low compliance during the trip. Another reason for failed protective effect of anti-malaria chemoprophylaxis against *P. ovale* infection is the ability of *P. ovale* to form hypnozoites, which can survive chemoprophylaxis [18].

In case A, there was a delay in malaria diagnosis. This case is a good example of clinical setting where malaria tends to be overlooked at the initial stage of medical investigation due to low level of suspicion. From the initial haematological investigation, all but one (platelet count) showed values within the normal range. In many hospital settings, especially those of urban areas and non-malaria endemic regions, such investigation outcome usually carries a thin possibility of instigating a blood film microscopic examination. Indeed, a case of traveller’s malaria that developed into severe complications due to delayed diagnosis was reported in Croatia recently [25]. The available reported malaria with delayed diagnoses may just be a tip of the iceberg; and such incident may happen more frequently than expected. Thus malaria should have been suspected if patient presented with fever, thrombocytopaenia, and showed history of travelling to malaria-endemic areas within the past one year. The long period between travel to malaria endemic country and disease onset may also contribute to delay in diagnosis, and subsequently the appropriate treatment. The late onset of symptoms due to long latency period provides challenges to clinical diagnoses upon admission. As mentioned previously, *P. ovale* spp. can form hypnozoites and result in long latency [18,26], which can be seen in the two cases presented here.

Patient B succumbed to the malaria infection and he did not have any medical problems prior to the illness. Patient A, who is a heavy drinker, was known to have hypertension even before the infection, survived. One possible explanation for this is the partial immunity gained by patient A from previous incidents of malaria. People who have past history of acquiring falciparum malaria and those staying in malaria-endemic regions are known to show less severe symptoms during subsequent malaria infections compared to people who are from non-malaria endemic regions (malaria-naïve) [27]. Indeed, such protective effect of acquired partial immunity via previous exposure to malaria was noted and mentioned in previous reports on falciparum and vivax malaria-related ARDS [5,28,29]. Furthermore, malaria related pulmonary complications are often seen in non-immune patients [30]. Patient A might be partially protected against severe complications due to previous exposure to malaria. Patient B did not have prior exposure to malaria, and therefore, he suffered and succumbed to the severe complications in his first attack of malaria infection. Even so, pathogenesis of ovale malaria is not intensively studied and protection against ovale malaria complications conferred by previous exposure has not been investigated. Therefore, more investigations are needed to validate this postulation.

One important note worth mentioning here is the trend of ARDS onset and development in this case. Similar to the situation of malaria-induced ARDS reported previously [10,31,32], patient B showed normal breathing without wheezing and crepitations upon admission, but his respiratory function deteriorated towards the grave complication of ARDS afterwards. Therefore, physicians should pay close attention to the respiratory condition of every admitted malaria case. Moreover, malaria is regarded as one of the important causes of ARDS [1].

In case B, nosocomial *Enterobacter cloacae* infection was diagnosed during the course of his hospital stay. Here, bacterial infection was ruled out as the primary cause of ARDS in patient B as the bacteriological culture showed positive results as late as day 15, whereas he showed symptoms of acute lung injury as early as day 4. Undoubtedly, the bacterial infection that occurred at such critical timing would worsen the health condition of the patient, which acted as the secondary contributing factor towards the fatal end for this case. Another point worth mentioning was the onset of the ARDS symptoms after the initiation of anti-malarial therapy, which was similar to many cases of malaria-associated ARDS reported previously [9,10,30-34]. The pulmonary injury seen in this case was likely to be a post-treatment related pulmonary inflammation. Nonetheless, a few case reports of malaria-induced ARDS with pre-treatment onset were described previously [5,35]. In addition, quinine was known to be capable of inducing pulmonary injury [34]. Hence, the quinine therapy that was briefly applied on the patient may contribute to the worsening of his lung injury. Nevertheless, quinine therapy was immediately replaced with artesunate within the same day.

Thrombocytopaenia was seen in both cases. Such phenomenon has been observed in patients infected with *P. falciparum*, *P. vivax*, and *P. knowlesi*[8,21,36-39]. Thrombocytopaenia may serve as a contributing factor, instead of a primary cause of malaria-related severe pulmonary injury. Indeed, the platelet reading of patient A (survived case) was lower than that of patient B (fatal case) upon admission. Other events that are believed to contribute towards acute pulmonary injuries include: host immunologic responses [34], rosetting phenomenon, which is found in all four species of human malaria parasites [40-44], and sequestration that is found in *P. falciparum* and to a lesser, but significant extent, *P. vivax*[45]. This event may account for the much higher prevalence of ARDS complications seen in falciparum malaria. Nonetheless, the pathophysiology of malaria-associated ARDS is not completely understood [1,10,30].

Another aspect of this fatal case of *P. ovale* infection that deserves attention is the acute renal failure. Acute renal failure is another potentially fatal complication in malaria [25,46,47]. However, such complication is reported mostly in falciparum malaria [47-49], the newly emerging knowlesi malaria [21,50], and occasionally, in a few cases of *P. vivax* and *P. malariae* infections [51,52]. A fatal case of vivax malaria with acute renal failure was reported recently [53]. This is by far the first reported case of *P. ovale* infection with acute renal injury. In case B, the patient suffered renal injury a few days before he came down with nosocomial sepsis. Therefore, the acute renal injury seen in this case was less likely to be caused by nosocomial sepsis. Nevertheless, the nosocomial sepsis might act as the secondary contributing factor towards the renal failure in this patient. Besides, the medications used on the patient such as vancomycin is potentially nephrotoxic [54], thus serving as another potential contributing factor that worsened the acute renal injury. Hence, such therapy was discontinued.

Apart from the differences in pathological development of both cases, the anti-malaria treatment regimes used in both cases were dissimilar as well. For patient A, Riamet® with primaquine was used whereas for patient B, chloroquine and primaquine were prescribed at the initial stage of treatment. Parasites were not detected from patient A’s blood smear in less than 48 hours after initiation of antimalarial treatment. On the other hand, complete parasite clearance could only be declared on patient B after switching the medication to quinine and artesunate. Consequently, the parasite clearance took as long as five days to accomplish. Parasite clearance was much faster in treatment strategy using the artemether-lumefantrine-primaquine combination than that of chloroquine-primaquine combination. Indeed, artemisinin-based combination therapy (ACT) has been proven to show high efficacy in treating falciparum malaria [55] and non-falciparum malaria [56]. The much slower parasite clearance by chloroquine-based treatment may be due to the stage-specific anti-malarial property of chloroquine. As shown by previous studies, the trophozoites of *P. vivax*, *P. malariae* and *P. ovale* are relatively insusceptible to chloroquine [57-59]. Therefore, ACT such as Riamet® can serve as an effective first line treatment even for non-falciparum malaria.

Severe ovale malaria is not commonly found. There are a few reports of *P. ovale* infection with ARDS [9-12], and one case of ovale malaria with splenic complication [60]. Fatal ovale malaria is even rarer. A fatal case of ovale malaria involving a young Moroccan soldier was reported recently [12]. Interestingly, this ovale malaria casualty also suffered ARDS complication. This recent case report and our report show that *P. ovale* is capable of causing severe complications and death. For that reason, patients with *P. ovale* infection should not be regarded as “ultimately benign” and taken lightly, particularly in the non-immune travellers. In view of this, travel history as well as past history of acquiring malaria in patients with fever must be recorded in detail to assist in the diagnosis and management of travellers’ malaria and severe malaria.

## Conclusion

Two imported cases of *P. ovale* infections with different pathological progress were reported. *Plasmodium ovale* is potentially capable of causing severe complications, if not death. In view of the differences between these two cases, complete clinical history of malaria patient, especially the travel history and history of malaria exposure are vital for successful treatment. Monitoring of respiratory and renal function of malaria patients, regardless of the species of malaria parasites involved is important during the course of hospital admission. In addition, ACT such as Riamet® should be applied to malaria patients regardless of the species of aetiological agents for prompt and efficient treatment.

## Competing interests

The authors declared that they have no competing interests.

## Authors’ contributions

WCL, YLL, and RM collected, analysed and interpreted the data. LHT, AK and SFSO collected blood samples, conducted clinical diagnoses and treatment intervention. FWC conducted and processed molecular diagnoses. MYF constructed and analysed phylogenetic tree. WCL, YLL, LHT and RM arranged the data, conceptualized and prepared the manuscript. All authors read and approved the final manuscript.

## References

[B1] Mohan A Sharma SK Bollineni S Acute lung injury and acute respiratory distress syndrome in malaria J Vector Borne Dis 2008 45 179 193 18807374

[B2] Lucas R Lou J Morel DR Ricou B Suter PM Grau GE TNF receptors in the microvascular pathology of acute respiratory distress syndrome and cerebral malaria J Leukoc Biol 1997 61 551 558 9129203 10.1002/jlb.61.5.551

[B3] Vásquez AM Tobón A Pathogenic mechanisms in *Plasmodium falciparum* malaria Biomedica 2012 32 Suppl 1 106 120 23235819 10.1590/S0120-41572012000500012

[B4] Deroost K Tyberghein A Lays N Noppen S Schwarzer E Vanstreels E Komuta M Prato M Lin JW Pamplona A Janse CJ Arese P Roskams T Daelemans D Opdenakker G Van den Steen PE Hemozoin induces lung inflammation and correlates with malaria-associated acute respiratory distress syndrome Am J Respir Cell Mol Biol 2013 48 589 600 10.1165/rcmb.2012-0450OC 23328641

[B5] Lomar AV Vidal JE Lomar FP Barbas CV de Matos GJ Boulos M Acute respiratory distress syndrome due to vivax malaria: case report and literature review Braz J Infect Dis 2005 9 425 430 16410895 10.1590/s1413-86702005000500011

[B6] Agarwal R Nath A Gupta D Noninvasive ventilation in *Plasmodium vivax* related ALI/ARDS Intern Med 2007 46 2007 2011 10.2169/internalmedicine.46.0401 18084125

[B7] Lozano F Leal M Lissen E Munoz J Bautista A Regordan C [*P. falciparum* and *P. malariae* malaria complicated by pulmonary edema with disseminated intravascular coagulation] (in French) Presse Med 1983 12 3004 3005 6228898

[B8] Daneshvar C Davis TM Cox-Singh J Rafa’ee MZ Zakaria SK Divis PC Singh B Clinical and laboratory features of human *Plasmodium knowlesi* infection Clin Infect Dis 2009 49 852 860 10.1086/605439 19635025 PMC2843824

[B9] Lee EY Maguire JH Acute pulmonary edema complicating ovale malaria Clin Infect Dis 1999 29 697 698 10.1086/598667 10530480

[B10] Rojo-Marcus G Cuadros-González J Mesa-Latorre JM Culebras-López AM de Pablo-Sánchez R Case report: acute respiratory distress syndrome in a case of *Plasmodium ovale* malaria Am J Trop Med Hyg 2008 79 391 393 18784231

[B11] Rozé B Lambert Y Gelin E Geffroy F Hutin P [*Plasmodium ovale* malaria severity] (in French) Med Mal Infect 2011 41 213 220 10.1016/j.medmal.2010.07.006 21194860

[B12] Hachimi MA Hatim EA Moudden MK Elkartouti A Errami M Louzi L Hanafi SM Mahmoudi A [The acute respiratory distress syndrome in malaria: is it always the prerogative of *Plasmodium falciparum*?] (in French) Rev Pneumol Clin 2013 S0761–8417 13 00071 10.1016/j.pneumo.2013.03.001 23688721

[B13] Collins WE Jeffery GM *Plasmodium ovale*: parasite and disease Clin Microbiol Rev 2005 18 570 581 10.1128/CMR.18.3.570-581.2005 16020691 PMC1195966

[B14] Snounou G Pinheiro L Antunes AM Ferreira C do Rosario VE Non-immune patients in the Democratic Republic of São Tomé e Principe reveal a high level of transmission of *P. ovale* and *P. vivax* despite low frequency in immune patients Acta Trop 1998 70 197 203 10.1016/S0001-706X(98)00021-7 9698266

[B15] Sutherland CJ Tanomsing N Nolder D Oguike M Jennison C Pukrittayakamee S Dolecek C Hien TT do Rosário VE Arez AP Pinto J Michon P Escalante AA Nosten F Burke M Lee R Blaze M Dan Otto T Barnwell JW Pain A Williams J White NJ Day NPJ Snounou G Lockhart PJ Chiodini PL Imwong M Polley SD Two nonrecombining sympatric forms of the human malaria parasite *Plasmodium ovale* occur globally J Infect Dis 2010 201 1544 1550 10.1086/652240 20380562

[B16] Sutherland CJ Polley SD Tibayrenc M Genomic Insights into the Past, Current and Future Evolution of Human Parasites of the Genus *Plasmodium* Genetics and Evolution of the Infectious Diseases 2011 London: Elsevier 607 635

[B17] Calderaro A Piccolo G Gorrini C Montecchini S Rossi S Medici MC Chezzi C Snounou G A new real-time PCR for the detection of *Plasmodium ovale wallikeri* PLoS One 2012 7 e48033 10.1371/journal.pone.0048033 23110165 PMC3480495

[B18] Nolder D Oguike MC Maxwell-Scott H Niyazi HA Smith V Chiodini PL Sutherland CJ An observational study of malaria in British travellers: *Plasmodium ovale wallikeri* and *Plasmodium ovale curtisi* differ significantly in the duration of latency BMJ Open 2013 3 e002711 10.1136/bmjopen-2013-002711 23793668 PMC3657643

[B19] Singh B Kim Sung L Matusop A Radhakrishnan A Shamsul SS Cox-Singh J Thomas A Conway DJ A large focus of naturally acquired *Plasmodium knowlesi* infections in human being Lancet 2004 363 1017 1024 10.1016/S0140-6736(04)15836-4 15051281

[B20] Cox-Singh J Davis TM Lee KS Shamsul SS Matusop A Ratnam S Rahman HA Conway DJ Singh B *Plasmodium knowlesi* in humans is widely distributed and potentially life threatening Clin Infect Dis 2008 46 165 171 10.1086/524888 18171245 PMC2533694

[B21] Lee WC Chin PW Lau YL Chin LC Fong MY Yap CJ Supramaniam RR Mahmud R Hyperparasitaemic human *Plasmodium knowlesi* infection with atypical morphology in peninsular Malaysia Malar J 2013 12 88 10.1186/1475-2875-12-88 23496970 PMC3600032

[B22] Basic Local Alignment Search Tool (BLAST) [http://blast.ncbi.nlm.nih.gov] 10.1101/pdb.top17 21357135

[B23] Tamura K Dudley J Nei M Kumar S MEGA 4: evolutionary genetics analysis (MEGA) software version 4.0 Mol Biol Evol 2007 24 1596 1599 10.1093/molbev/msm092 17488738

[B24] Lim YA Mahmud R Chew CH Thiruventhiran T Chua KH *Plasmodium ovale* infection in Malaysia: first imported case Malar J 2010 9 272 10.1186/1475-2875-9-272 20929588 PMC2959071

[B25] Troselj-Vukić B Vuksanović-Mikulicić S Sladoje-Martinović B Milotić I Slavuljica I Unrecognized malaria and its consequences: a case report of severe malaria with acute renal failure Coll Antropol 2013 37 611 613 23941012

[B26] Miller MJ Marcus DM Cameron DG Latent infections with *Plasmodium ovale* malaria Canad Med Ass J 1965 92 1241 1247 14296004 PMC1928406

[B27] Høgh B Clinical and parasitological studies on immunity to *Plasmodium falciparum* malaria in children Scand J Infect Dis Suppl 1996 102 1 53 9060051

[B28] Tanios MA Kogelman L McGovern B Hassoun PM Acute respiratory distress syndrome complicating *Plasmodium vivax* malaria Crit Care Med 2001 29 665 667 10.1097/00003246-200103000-00037 11373440

[B29] Sarkar S Saha K Das CS Three cases of ARDS: an emerging complication of *Plasmodium vivax* malaria Lung India 2010 27 154 157 10.4103/0970-2113.68323 20931035 PMC2946718

[B30] Taylor WR Cañon V White NJ Pulmonary manifestations of malaria: recognition and management Treat Respir Med 2006 5 419 428 10.2165/00151829-200605060-00007 17154671

[B31] Anstey NM Jacups SP Cain T Pearson T Ziesing PJ Fisher DA Currie BJ Marks PJ Maguire GP Pulmonary manifestations of uncomplicated falciparum and vivax malaria: cough, small airways obstruction, impaired gas transfer, and increased pulmonary phagocytic activity J Infect Dis 2002 185 1326 1334 10.1086/339885 12001051

[B32] Price L Planche T Rayner C Krishna S Acute respiratory distress syndrome in *Plasmodium vivax* malaria: case report and review of the literature Trans R Soc Trop Med Hyg 2007 101 655 659 10.1016/j.trstmh.2007.02.014 17433389

[B33] Martell RW Kallenbach J Zwi S Pulmonary oedema in the falciparum malaria Br Med J 1979 1 1763 1764 380762 10.1136/bmj.1.6180.1763-a PMC1599415

[B34] Taylor WR White NJ Malaria and the lung Clin Chest Med 2002 23 457 468 10.1016/S0272-5231(02)00004-7 12092039

[B35] Rahman AK Sulaiman FN *Plasmodium vivax* malaria presenting as acute respiratory distress syndrome: a case report Trop Doct 2013 43 83 85 10.1177/0049475513485733 23796679

[B36] Kakar A Bhoi S Prakash V Kakar S Profound thrombocytopenia in *Plasmodium vivax* malaria Diag Microbiol Infect Dis 1999 35 234 244 10.1016/s0732-8893(99)00069-3 10626136

[B37] Ansari S Khoharo HK Abro A Akhund IA Qureshi F Thrombocytopenia in *Plasmodium falciparum* malaria J Ayub Med Coll Abbottabad 2009 21 145 147 20524493

[B38] Thapa R Biswas B Mallick D Sardar S Modak S Childhood *Plasmodium vivax* malaria with severe thrombocytopenia and bleeding manifestations J Pediatr Hematol Oncol 2009 31 758 759 10.1097/MPH.0b013e3181b7eb12 19779377

[B39] Lacerda MV Mourão MP Coelho HC Santos JB Thrombocytopenia in malaria: who cares? Mem Inst Oswaldo Cruz 2011 106 Suppl 1 52 63 21881757 10.1590/s0074-02762011000900007

[B40] David PH Handunnetti SM Leech JH Gamage P Mendis KN Rosetting: a new cytoadherence property of malaria-infected erythrocytes Am J Trop Med Hyg 1988 38 289 297 3354764 10.4269/ajtmh.1988.38.289

[B41] Handunnetti SM David PH Perera KL Mendis KN Uninfected erythrocytes form “rosettes” around *Plasmodium falciparum* infected erythrocytes Am J Trop Med Hyg 1989 40 115 118 2645800 10.4269/ajtmh.1989.40.115

[B42] Udomsangpetch R Thanikkul K Pukrittayakamee S White NJ Rosette formation by *Plasmodium vivax* Trans R Soc Trop Med Hyg 1995 89 635 637 10.1016/0035-9203(95)90422-0 8594679

[B43] Angus BJ Thanikkul K Silamut K White NJ Udomsangpetch R Short report: rosette formation in *P. ovale* infection Am J Trop Med Hyg 1996 55 560 561 8940990 10.4269/ajtmh.1996.55.560

[B44] Lowe BS Mosobo M Bull PC All four species of human malaria parasites form rosettes Trans R Soc Trop Med Hyg 1998 92 526 10.1016/S0035-9203(98)90901-4 9861369

[B45] Carvalho BO Lopes SC Mogueira PA Orlandi PP Bargieri DY Blanco YC Mamomi R Leite JA Rodrigues MM Araújo MO Russell B Suwanarusk R Snounou G Rénia L Costa FT On the cytoadhesion of *Plasmodium vivax*-infected erythrocytes J infect Dis 2010 202 638 647 10.1086/654815 20617923

[B46] Trampuz A Jereb M Muzlovic I Prabhu RM Clinical review: severe malaria Crit Care 2003 7 315 323 10.1186/cc2183 12930555 PMC270697

[B47] Das BS Renal failure in malaria J Vector Borne Dis 2008 45 83 97 18592837

[B48] Eiam-Ong S Sitprija V Falciparum malaria and the kidney: a model of inflammation Am J Kidney Dis 1998 32 361 375 10.1053/ajkd.1998.v32.pm9740151 9740151

[B49] Sinniah R Lye W Acute renal failure from myoglobinuria secondary to myositis from severe falciparum malaria Am J Nephrol 2000 20 339 343 10.1159/000013611 10970990

[B50] Singh B Daneshvar C Human infections and detection of *Plasmodium knowlesi* Clin Microbiol Rev 2013 26 165 184 10.1128/CMR.00079-12 23554413 PMC3623376

[B51] Prakash J Singh AK Kumar NS Saxena RK Acute renal failure in *Plasmodium vivax* malaria J Assoc Physicians India 2003 51 265 267 12839348

[B52] Hendrickse RG Adeniyi A Edington GM Glasgow EF White RH Houba V Quartan malarial nephrotic syndrome. Collaborative clinicopathological study in Nigerian children Lancet 1972 1 1143 1149 4113056 10.1016/s0140-6736(72)91373-6

[B53] Patel MP Kute VB Gumber MR Gera DN Shah PR Patel HV Trivedi HL Vanikar AV An unusual case of *Plasmodium vivax* malaria monoinfection associated with crescentic glomerulonephritis: a need for vigilance Parasitol Res 2013 112 427 430 10.1007/s00436-012-3040-5 22806325

[B54] Gupta A Biyani M Khaira A Vancomycin nephrotoxicity: myths and facts Neth J Med 2011 69 379 383 21978980

[B55] Bhattarai A Ali AS Kachur SP Mártensson A Abbas AK Khatib R Al-mafazy A Ramsan G Rotliant G Gerstenmaier JF Molteni F Abdulla S Montgomery SM Kaneko A Björkman A Impact of artemisinin-based combination therapy and insecticide-treated nets on malaria burden in Znazibar PLoS Med 2007 4 e309 10.1371/journal.pmed.0040309 17988171 PMC2062481

[B56] Mombo-Ngoma G Kleine C Basra A Wűrbel H Diop DA Capan M Adegnika AA Kurth F Mordműller B Joanny F Kremsner PG Ramharter M Bélard S Prospective evaluation of artemether-lumefantrine for the treatment of non-falciparum and mixed-species malaria in Gabon Malar J 2012 11 120 10.1186/1475-2875-11-120 22515681 PMC3393621

[B57] Russell B Chalfein F Prasetyorini B Kenangalem E Piera K Suwanarusk R Brockman A Prayoga P Sugiarto P Cheng Q Tjitra E Anstey NM Price RN Determinants of in vitro drug susceptibility testing of *Plasmodium vivax* Antimicrob Agents Chemother 2008 52 1040 1045 10.1128/AAC.01334-07 18180357 PMC2258486

[B58] Sharrock WW Suwanarusk R Lek-Uthai U Edstein MD Kosaisavee V Travers T Jaidee A Sriprawat K Price RN Nosten F Russell B *Plasmodium vivax* trophozoites insensitive to chloroquine Malar J 2008 7 94 10.1186/1475-2875-7-94 18505560 PMC2430579

[B59] Siswantoro H Russell B Ratcliff A Prasetyorini B Chalfein F Marfurt J Kenangalem E Wuwung M Piera KA Ebsworth EP Anstey NM Tjitra E Price RN In vivo and in vitro efficacy of chloroquine against *Plasmodium malariae* and *P. ovale* in Papua, Indonesia Antimicrob Agents Chemother 2011 55 197 202 10.1128/AAC.01122-10 20937779 PMC3019630

[B60] Cinquetti G Banal F Rondel C Plancade D de Saint Roman C Adriamanantena D Ragot C Védy C Graffin B Splenic infarction during *Plasmodium ovale* acute malaria: first case reported Malar J 2010 9 288 10.1186/1475-2875-9-288 20955610 PMC2984568

